# Chemoport-related right innominate vein stenosis in patients with colorectal cancer: A retrospective risk factor analysis

**DOI:** 10.1371/journal.pone.0348521

**Published:** 2026-05-20

**Authors:** Junghoon Kim, Jeong Sub Lee, Choong Guen Chee, Jihoon Woo, Youngjune Kim, Eugene Lee, Joon Woo Lee, Sang Il Choi

**Affiliations:** 1 Department of Radiology, Seoul National University Bundang Hospital, Seongnam-si, Gyeonggi-do, Republic of Korea; 2 Seoul National University College of Medicine, Seoul, Republic of Korea; 3 Gold-man Hospital, Seoul, Republic of Korea; Stanford University School of Medicine, UNITED STATES OF AMERICA

## Abstract

Little information is available regarding the factors that contribute to the occurrence of right innominate vein stenosis in patients with chemoports. Thus, the aims of this study were to evaluate the prevalence of chemoport-related right innominate vein stenosis (CR-RIVS) in patients with colorectal cancer and to identify factors associated with increased CR-RIVS risk. This retrospective study included patients with colorectal cancer (age, ≥ 19 years) who underwent their first chemoport insertion via the right internal jugular vein between August 2015 and August 2020. The prevalence of CR-RIVS was evaluated using follow-up contrast-enhanced chest computed tomography post-chemoport insertion. Risk factors were analyzed using Classification and Regression Tree, Kaplan-Meier survival, and Cox regression analyses. A subgroup analysis was also conducted among patients in palliative care to assess the impact of bevacizumab and cetuximab treatment on the prevalence of CR-RIVS. Among 1,442 patients with colorectal cancer (mean age, 62.1 ± 11.8 years; 850 males), the prevalence of CR-RIVS was 6.6%. The median time from chemoport insertion to CR-RIVS occurrence was 405 days (interquartile range, 256–908 days). The Classification and Regression Tree analysis identified palliative settings as the key predictor of CR-RIVS. Age, palliative settings, and a time×palliative setting interaction were identified as statistically significant variables (*p* < 0.001). In the 728 palliative patients, bevacizumab treatment was significantly associated with a higher risk of CR-RIVS (hazard ratio = 1.51, 95% confidence interval: 1.25–1.82, *P* < 0.001), whereas cetuximab was not (*p* = 0.85). The CR-RIVS-free probability was lower in patients receiving palliative chemotherapy than in those undergoing adjuvant chemotherapy (*p* < 0.001); it was also lower in the bevacizumab-treated patients (*p* < 0.001) in the palliative setting subgroup analysis. In summary, the prevalence of CR-RIVS was 6.6% in patients with colorectal cancer. Bevacizumab use showed the strongest association with CR-RIVS occurrence in the palliative subgroup analysis.

## Introduction

Implantable venous ports (chemoports) are widely used for the safe and efficient delivery of chemotherapeutic agents to patients with cancer. Despite their utility, chemoports are associated with potential complications, including thrombotic events such as catheter-related thrombosis and venous stenosis. These thrombotic complications can arise from trauma to the vessel wall, which can lead to stenosis or occlusion of the accessed vein, or thrombus formation around the catheter tip, thereby compromising venous flow [1–4].

The incidence of venous stenosis in patients with peripherally inserted central catheters or chemoports has been reported to be approximately 7% [5]. Venous stenosis, particularly that involving the innominate vein, can lead to potentially severe clinical issues such as face and arm swelling, jugular venous distension, superior vena cava syndrome, and vascular access failure [6]. Furthermore, central venous stenosis can negatively affect the quality of diagnostic imaging, particularly contrast-enhanced computed tomography (CT) and magnetic resonance angiography, by increasing perivenous artifacts and inducing venous reflux [7,8]. These imaging artifacts can compromise the quality of diagnostic evaluations, making it more challenging to accurately assess abnormalities in vascular and adjacent structures. Contrast reflux into the vertebral body may also lead radiologists to misinterpret the findings as sclerotic metastasis, also known as vanishing bone metastasis syndrome, potentially resulting in harm or incurring additional costs to the patient [9].

To the best of our knowledge, no studies have comprehensively evaluated the factors that contribute to the occurrence of right innominate vein stenosis (RIVS) in patients with chemoports. Considering that venous stenosis can develop through partial lysis and recanalization following venous thrombosis [10], potential associations may exist between the occurrence of innominate vein stenosis and the known risk factors for chemoport-induced thrombosis, which include prior chemoport placement, a history of catheter-related thrombosis, repeated insertion attempts, specific catheter characteristics (e.g., material composition and tip positioning), and certain chemotherapy regimens, such as those involving bevacizumab, in patients with colorectal cancer (CRC) [2,11–13]. More specifically, the administration of bevacizumab has been shown to be significantly associated with an increased risk of chemoport-related thrombosis in patients with CRC and vertebral venous congestion syndrome [9,14].

Thus, the aims of this study were to assess the prevalence of chemoport-related RIVS (CR-RIVS) in patients with CRC and to identify factors associated with increased CR-RIVS risk.

## Materials and methods

### Study design and ethical approval

This single-center, retrospective, cross-sectional, multi-reader study was conducted in accordance with the tenets of the Helsinki Declaration and received approval from the Institutional Review Board of Seoul National University Bundang Hospital (IRB No. B-2402-881-104) on January 30, 2024. The requirement for written or verbal informed consent was waived by the Institutional Review Board of Seoul National University Bundang Hospital owing to the retrospective nature of the study and the use of anonymized data.

For this retrospective study, patient data were accessed between February 1, 2024, and January 24, 2025. Information that could identify individual participants was accessible to the authors during data extraction for verification purposes. After data collection was completed, all datasets were fully anonymized prior to analysis.

### Study participants

**Fig 1:**
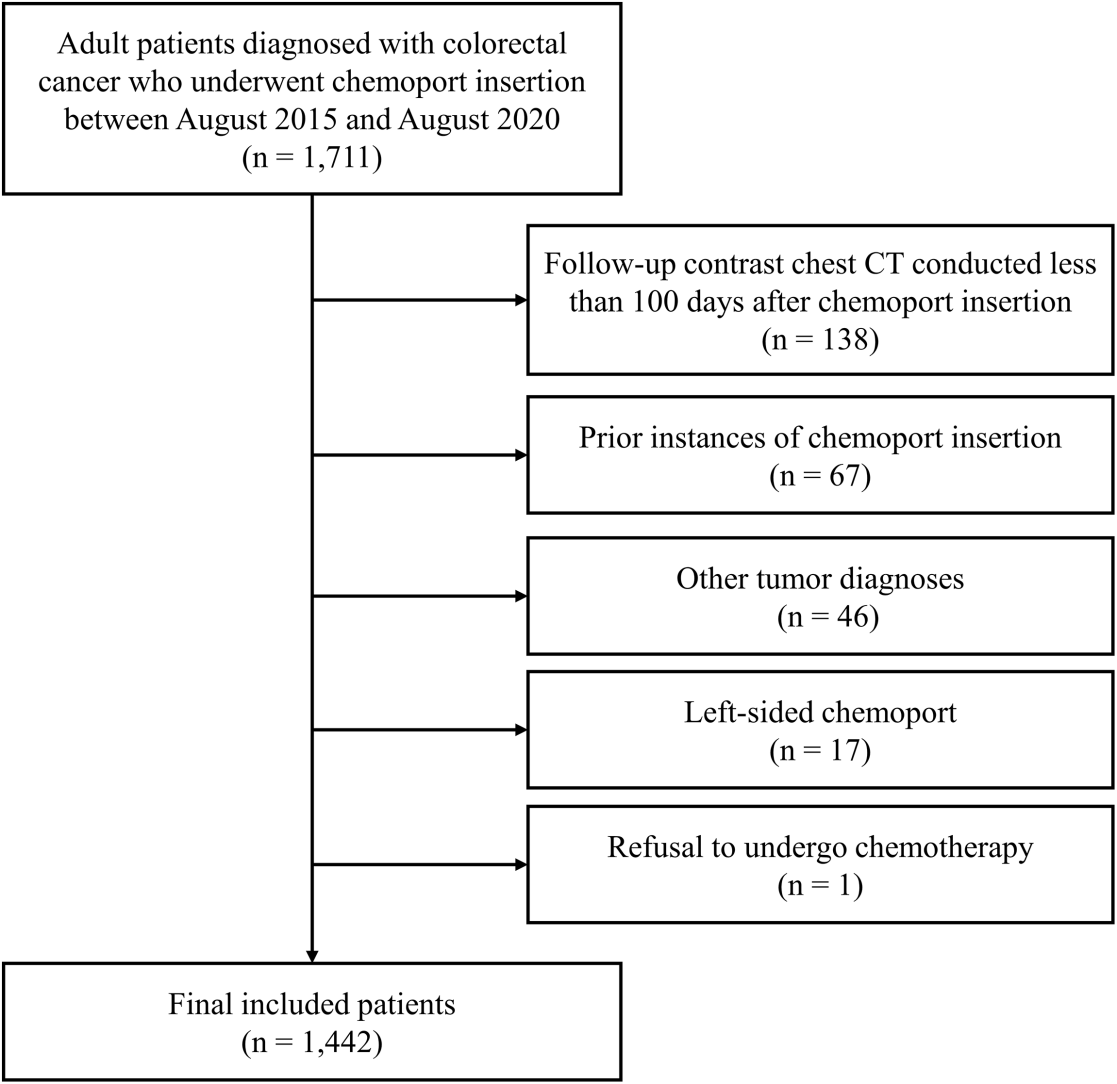
Flowchart of patient selection. CT, computed tomography.

All consecutive patients with a colorectal tumor diagnosis, aged 19 years or older, who underwent their first chemoport insertion at our institution between August 2015 and August 2020 and received at least one follow-up contrast-enhanced chest CT scan post-chemoport insertion were included in the study. The exclusion criteria were as follows: i) patients whose last follow-up contrast-enhanced chest CT scans were conducted within 100 days after chemoport insertion (n = 138); ii) patients who underwent a chemoport insertion after already having one installed and removed previously (n = 67); iii) patients diagnosed with cancers other than colorectal adenocarcinoma (n = 46); iv) patients with left-sided chemoport insertion (n = 17); and v) patients who refused to undergo chemotherapy after chemoport insertion (n = 1). After applying these criteria, 1,442 patients were included in this study (Fig 1). The first exclusion criterion was based on the results of a previous study that reported an interquartile range (IQR) of 204–881 days for the time interval between chemoport insertion and the occurrence of vertebral venous congestion to consider the need for a minimal observation period for venous congestion to occur [9].

Data pertaining to clinical variables such as age, sex, body mass index (BMI), smoking history (pack-years), underlying disease (hypertension, diabetes mellitus, dyslipidemia, and proteinuria), a history of thromboembolic events during the chemotherapy period, cancer type, cancer stage, specific chemotherapy regimens administered, and a history of central line insertion were collected from the patients’ electronic medical records.

### Image analysis

**Fig 2:**
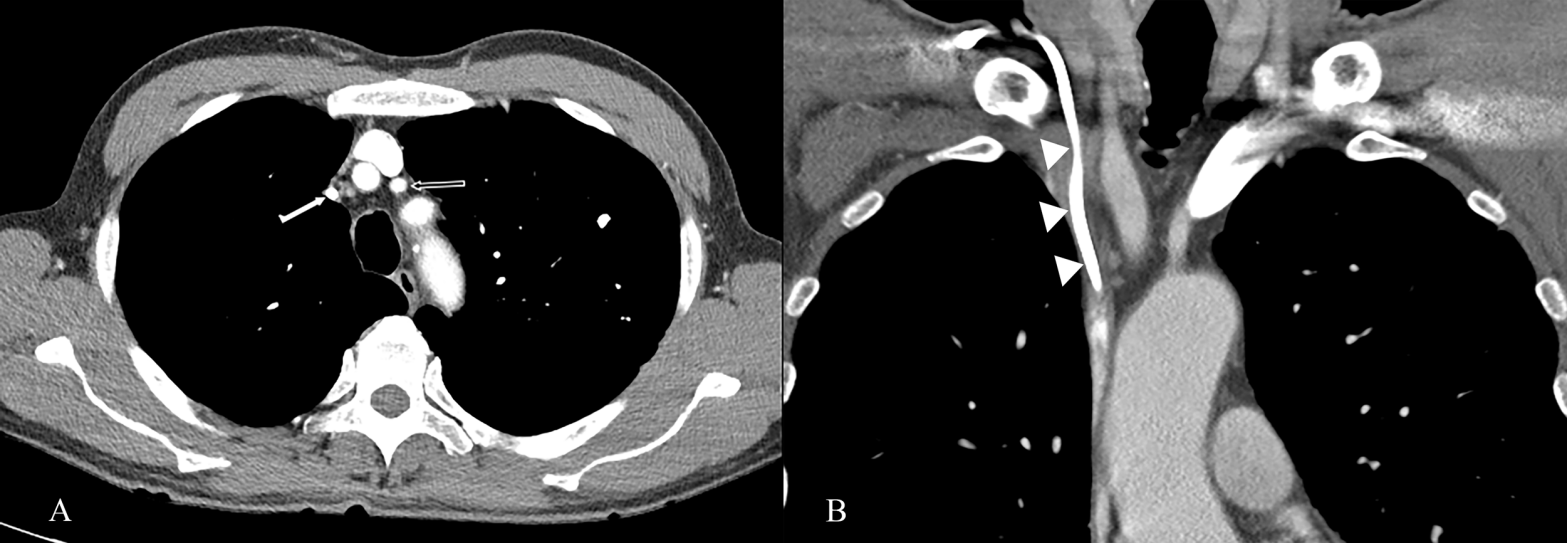
Representative images from a 66-year-old male patient diagnosed with sigmoid colon cancer who experienced CR-RIVS. **a)** Axial contrast-enhanced CT scan showing the diminished cross-sectional area of the right innominate vein (white arrow) compared to that of the left carotid artery (grey arrow). **b)**: Contrast-enhanced CT scan in the coronal plane showing long segmental right innominate stenosis along the catheter (arrowheads). CR-RIVS, chemoport-related right innominate vein stenosis; CT, computed tomography.

RIVS was defined as a concentric reduction in the cross-sectional area of the right innominate vein on contrast-enhanced chest CT equal to or smaller than that of the proximal left common carotid artery (Fig 2). Only the right innominate vein was chosen for image analysis because the right side is favored over the left as the initial site of chemoport insertion. Furthermore, the decision to focus on the right innominate vein was influenced by the possibility of physiologic compression of the left innominate vein between the sternum and aortic arch [15,16], as well as the difficulty of assessing the cross-sectional area of the horizontally running anatomical structure on axial images. After participating in an educational session that discussed representative cases, two radiologists with 12 years of experience (J.K. and C.G.C.) and two clinical researchers (J.S.L. and J.H.W.) dedicated to the field of radiology assessed the presence of RIVS. A consensus reading was performed in questionable cases.

### Statistical analysis

The required number of patients in this study was calculated based on the prevalence of innominate venous stenosis of 8.2% (95% confidence interval (CI): 6.6%–10.0%) in patients with chemoports reported by Shin et al. [9]. At least 1,500 patients were needed to ensure for that more than 100 participants with CR-RIVS would be included in the multivariable analysis for approximately 10 variables [17], using the lower limit of the previously reported prevalence (6.6%). The prevalence of RIVS in these patients was calculated and reported with 95% CIs. Descriptive statistics of the included patients are presented as means ± standard deviations (SDs) for continuous variables or as frequencies and percentages for categorical variables. Of the 1,442 patients, 71 had missing values (4.9%); therefore, statistical analyses were conducted on the patients with complete datasets [18]. The decision tree model for predicting CR-RIVS was generated in the Classification and Regression Tree (CART) analysis using “cpart” commands from the “party” package in R software. Potential risk factors included in the analysis were age, sex, BMI, smoking history, hypertension, diabetes, dyslipidemia, proteinuria, thromboembolism, palliative treatment, previous history of central line insertion, and the chemoport dwell time. Age, smoking history, and the chemoport dwell time were treated as continuous variables, whereas the others were treated as categorical variables throughout the analyses. BMI was categorized as underweight (< 18.5 kg/m^2^), normal (18.5–24.9 kg/m^2^), or other (≥ 25.0 kg/m^2^).

Univariable Cox regression analyses were performed for variables that were potentially associated with CR-RIVS; those with a P*-*value < 0.1 were selected for inclusion in the subsequent multivariable Cox regression model. The Schoenfeld residuals were used to assess any time-dependent interactions between variables; those with significant time-dependent interactions were incorporated into the multivariable Cox regression model. For each model, goodness of fit was assessed using the likelihood ratio, Wald, and score (log-rank) tests. Model discrimination was evaluated using the concordance index (C-index).

Kaplan-Meier survival analysis was conducted using log-rank tests, and CR-RIVS-free survival analysis was conducted to compare outcomes between patients in adjuvant and palliative treatment settings. Patients who did not experience venous stenosis before the study’s observational period were censored on the date of their latest contrast-enhanced chest CT scan. A subgroup analysis was performed on the patients who received treatment in a palliative setting to compare the outcomes of bevacizumab and cetuximab use. *P* < 0.05 was considered statistically significant.

R version 4.3.3 (R Foundation for Statistical Computing) and STATA (version 16; StataCorp) were used for all statistical analyses.

## Results

### Patient characteristics and CR-RIVS occurrence

**Fig 3:**
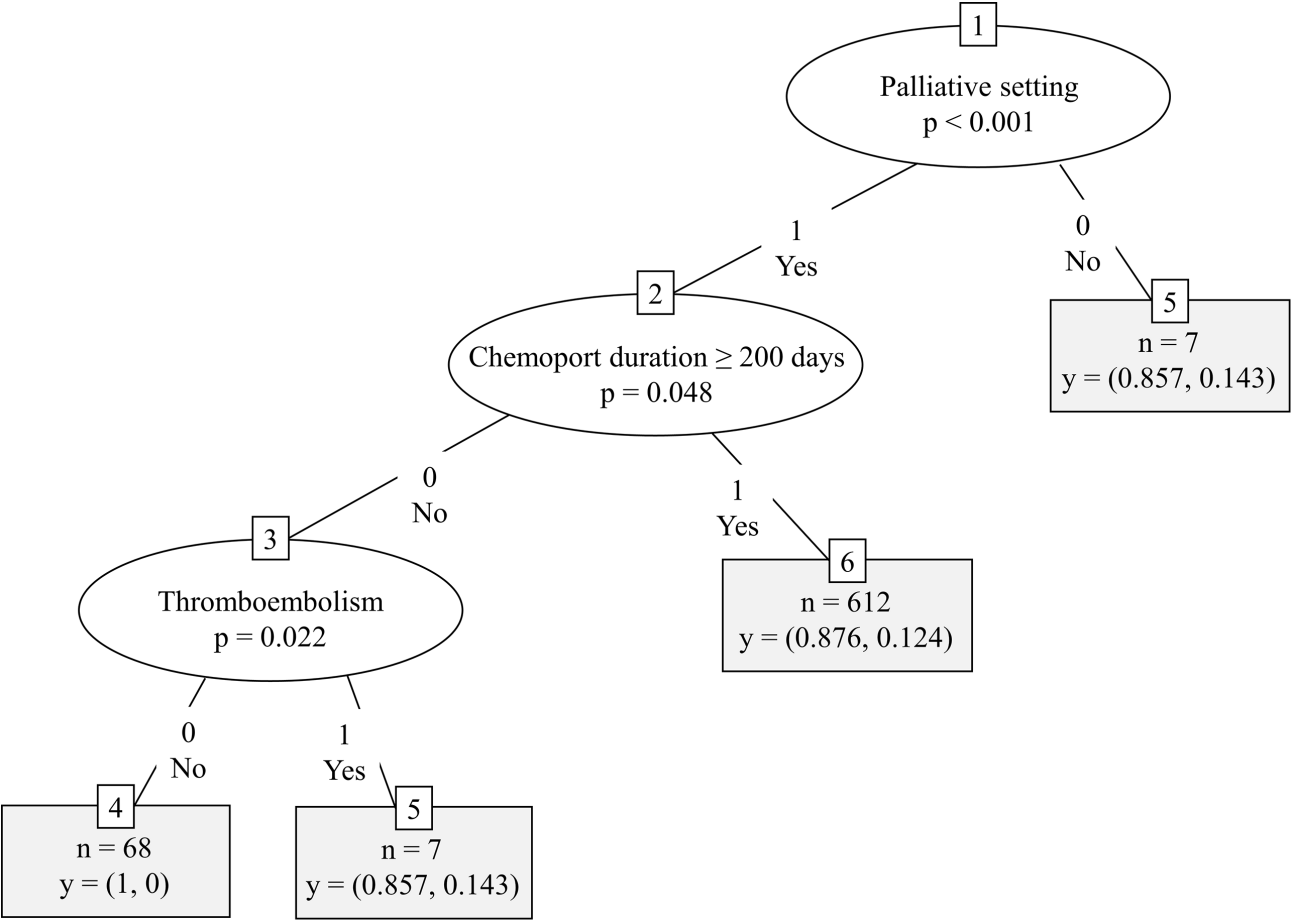
Density plots of CR-RIVS events. **a)** Density of events over time. **b)** Density of events across bevacizumab treatment cycles. CR-RIVS, chemoport-related right innominate vein stenosis.

A total of 1,442 eligible patients with CRC (mean age ± SD, 62.1 ± 11.8 years; 850 males) who received chemotherapy through a chemoport inserted via the right internal jugular vein and had adequate follow-up contrast-enhanced chest CT imaging were included in the analysis. Table 1 summarizes the demographic characteristics of the included patients. The prevalence of CR-RIVS was 6.6% (95/1,442 patients; 95% CI: 5.4%–8.0%), and the median time interval between the date of the initial chemoport insertion and CR-RIVS occurrence was 405 days (IQR: 256–908 days). Among the 95 patients with CR-RIVS, 10 (10.5%) developed port-related complications necessitating chemoport removal or exchange, including catheter-related thrombosis (n = 7) and port malfunction (n = 3). Fig 3 shows the density plots for the time interval between chemoport insertion and CR-RIVS events (Fig 3a) and the number of bevacizumab cycles (Fig 3b) administered prior to the CR-RIVS event. The median observation period (the interval between the initial chemoport insertion and the latest chest CT examination) was 1,405 days (IQR: 826–1,814 days), and the total dwell time of the chemoport during the observation period was 227 days (IQR: 189–626 days). Of the included patients, 50.4% (n = 728) received palliative treatment; among these patients, 558 (76.6%) were treated with bevacizumab, 164 (22.5%) received cetuximab, 104 (14.3%) were administered both bevacizumab and cetuximab, and 110 (15.1%) received neither drug during palliative care.

**Table 1:** Participants’ baseline demographic and clinical characteristics.

Characteristics	All (n = 1,442)	Adjuvant setting (n = 714)	Palliative setting (n = 728)
**Age, years**	62.1 ± 11.8	62.2 ± 11.3	62.0 ± 12.2
**Sex**			
**Male**	850 (59.0%)	419 (58.7%)	431 (59.2%)
^a^ **Body mass index, kg/m**^**2**^			
**Underweight (<18.5)**	79 (5.5%)	20 (2.8%)	59 (8.1%)
**Normal (18.5–24.9)**	891 (61.8%)	438 (61.3%)	453 (62.2%)
**Overweight or Obese (≥ 25.0)**	469 (32.5%)	255 (35.7%)	214 (29.4%)
^**b**^ **Smoking history, pack-years**	8.8 ± 14.9	8.8 ± 14.2	8.9 ± 15.5
**Previous history/complications**			
^**c**^ **Hypertension**	506 (35.1%)	246 (34.4%)	260 (35.7%)
^**c**^ **Diabetes mellitus**	240 (16.6%)	114 (16.0%)	126 (17.3%)
^**c**^ **Dyslipidemia**	229 (15.9%)	119 (16.7%)	110 (15.1%)
^**d**^ **Dip stick test abnormality**	275 (19.1%)	104 (14.6%)	171 (23.5%)
**Thromboembolic event** **Cancer**	101 (7.0%)	14 (2.0%)	87 (12.0%)
**Colon cancer**	995 (69.0%)	492 (68.9%)	503 (69.1%)
**Rectal cancer**	447 (31.0%)	222 (31.1%)	225 (30.9%)
**Adjuvant chemotherapy**			
^**e**^ **FOLFOX**	–	639 (89.5%)	–
**Others**	–	75 (10.5%)	–
**First line palliative chemotherapy regimen**			
**Bevacizumab–FOLFOX**	–	–	253 (34.8%)
**Bevacizumab–FOLFIRI**^**f**^	–	–	203 (27.9%)
**Cetuximab–FOLFIRI**	–	–	108 (14.8%)
**Cetuximab–FOLFOX**	–	–	17 (2.3%)
**FOLFOX**	–	–	85 (11.7%)
**FOLFIRI**	–	–	21 (2.9%)
**Others**	–	–	16 (2.2%)
**None**	–	–	25 (3.4%)
**Overall bevacizumab usage**	–	–	170 (23.4%)
**Overall cetuximab usage**	–	–	164 (22.5%)
**Previous central line insertion**	100 (6.9%)	67 (9.4%)	33 (4.5%)
**Right innominate vein stenosis**	95 (6.6%)	14 (2.0%)	81 (11.1%)

Note: Data are presented as either numbers (%) or means ± standard deviations.

^a^Number of missing values: three.

^b^Number of missing values: 14.

^c^Number of missing values: eight.

^d^Number of missing values: 51.

^e^FOLFOX, folinic acid, fluorouracil, and oxaliplatin regimen.

^f^FOLFIRI, folinic acid, fluorouracil, and irinotecan regimen.

### Identification of risk factors based on the decision tree and Cox regression models

**Fig 4:**
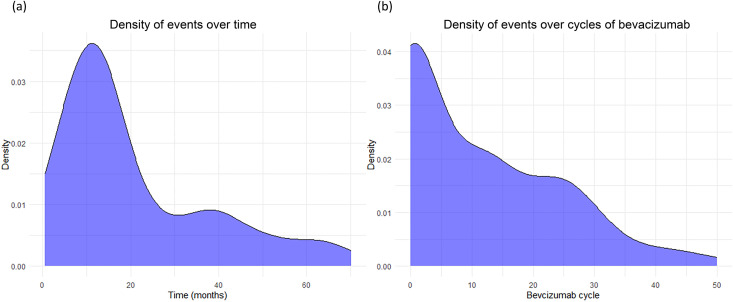
CART decision tree model for CR-RIVS. CART, Classification and Regression Tree; CR-RIVS, chemoport-related right innominate vein stenosis‌‌.

In the CART decision tree analysis, receiving palliative treatment (*p* < 0.001) was the first partitioning predictor in the model (Fig 4). The subsequent branching node was based on whether the patient had a chemoport dwell time of ≥ 200 days (*p* = 0.048), and the last branching node was based on whether the patient experienced a thromboembolism during the observation period (*p* = 0.022). The highest probability of venous stenosis was 14.3% in patients receiving palliative treatment who had a chemoport dwell time of less than 200 days and experienced thromboembolic events during the observation period.

Univariable Cox proportional hazards regression models were used to identify potential risk factors, including age, sex, BMI, smoking status, treatment in a palliative setting, underlying disease (hypertension, diabetes mellitus, dyslipidemia, and proteinuria), thromboembolic events experienced during the observation period, history of central line insertion, and chemoport dwell time. Age, BMI, hypertension, dyslipidemia, proteinuria, and treatment in a palliative setting were identified as statistically significant variables (Table 2). After adjusting for the violation of the assumption of the proportional hazards model for the palliative setting variable (*p* < 0.001), a multivariable Cox proportional hazards regression model was constructed that incorporated the interaction between time and treatment in the palliative setting (Table 3). Age, the palliative setting, and the interaction between time and the palliative setting were statistically significant variables (*p* < 0.001), with the palliative setting exhibiting decreasing hazard ratios (HRs) across time. In the subgroup Cox regression analysis of patients receiving palliative care, bevacizumab treatment was significantly associated with CR-RIVS occurrence, whereas cetuximab treatment was not (for bevacizumab: HR = 1.51, 95% CI: 1.25–1.82, *p* < 0.001; for cetuximab: HR = 1.02, 95% CI: 0.85–1.23, *p* = 0.85).

**Table 2:** Univariable Cox analysis for risk factors of chemoport-related right innominate venous stenosis.

Characteristics	Univariable analysis	
	Hazard Ratio (95% CI^a^)	P-value
**Age**	1.02 (1.01–1.02)	**<0.001**
**Sex (female)**	0.95 (0.85–1.07)	0.4
**Body Mass Index**		
**Underweight (<18.5)**	reference	
**Normal (18.5–24.9)**	0.81 (0.63–1.04)	0.1
**Overweight or Obese (≥ 25.0)**	0.74 (0.57–0.96)	**0.02**
**Smoking history, pack-years**	1.00 (0.997–1.00)	0.83
**Underlying diseases**		
**Hypertension**	1.13 (1.01–1.27)	**0.034**
**Diabetes Mellitus**	1.11 (0.96–1.29)	0.15
**Dyslipidaemia**	1.15 (0.99–1.34)	0.07
**Proteinuria**	1.51 (1.25–1.82)	**<0.001**
**Thromboembolic event**	0.98 (0.783–1.23)	0.88
**Cancer type**		
**Colon cancer**	reference	0.51
**Rectal cancer**	1.04 (0.92–1.17)	
**Previous central line insertion history**	1.01 (0.82–1.25)	0.9
**Palliative treatment**	1.77 (1.59–1.98)	**<0.001**

^a^CI = confidence interval.

**Table 3:** Multivariable Cox proportional hazards regression analysis of risk factors associated with chemoport-related right innominate venous stenosis.

Variables	Hazard Ratio (95% CI^a^)	P-value
**Age**	1.00 (1.00–1.01)	**0.002**
**Body mass index**		
**Underweight (<18.5 kg/m**^**2**^)	Reference	
**Normal (18.5–24.9 kg/m**^**2**^)	0.90 (0.70–1.12)	0.43
**Overweight or obese (≥ 25.0 kg/m**^**2**^)	0.83 (0.64–1.09)	0.18
**Hypertension**	0.99 (0.87–1.13)	0.93
**Dyslipidemia**	1.09 (0.93–1.27)	0.29
**Proteinuria**	1.05 (0.87–1.27)	0.64
**Palliative setting**	2.76 × 10^10^ (5.89 × 10^9^–1.23 × 10^11^)	**<0.001**
**Time × Palliative setting**	2.82 × 10^−3^ (1.92 × 10^−3^–4.15 × 10^−3^)	**<0.001**

^a^CI = confidence interval.

The palliative setting was the strongest predictor of CR-RIVS occurrence (HR = 1.771; 95% CI, 1.585–1.978; likelihood ratio χ² = 100.3; C-index = 0.616; p < 0.001). Proteinuria, age, and hypertension were also significantly associated with development of CR-RIVS, with hazard ratios of 1.509, 1.017 (per year), and 1.133; likelihood ratio χ² values of 16.7, 43.8, and 4.46; and C-indices of 0.521, 0.557, and 0.518, respectively.

### CR-RIVS-free survival analysis

**Fig 5:**
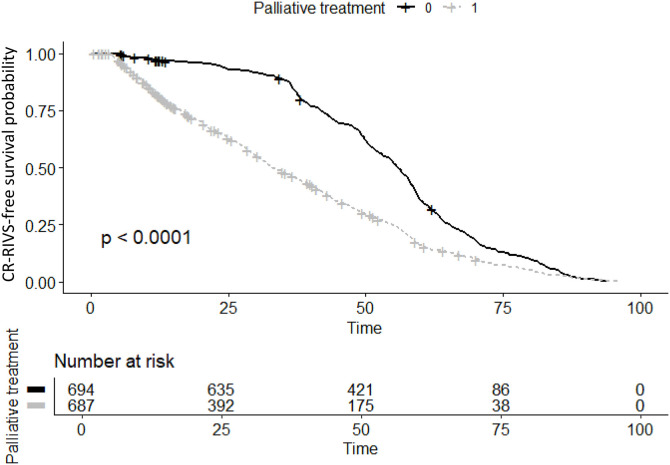
CR-RIVS-free survival analysis based on the nature of treatment (0): Patients receiving adjuvant therapy. (1): Patients receiving palliative treatment. The upper graph shows the survival probability over time, with the numbers at risk shown below it. CR-RIVS, chemoport-related right innominate vein stenosis.

**Fig 6:**
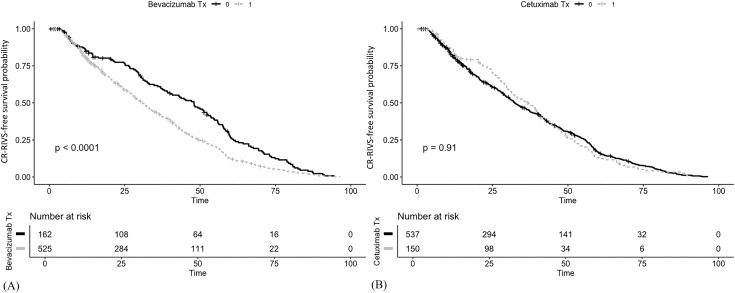
CR-RIVS-free survival analysis based on the chemotherapeutic agent administered. **a)** CR-RIVS-free probability over time and the number at risk in the palliative care patients treated with bevacizumab. **b)** CR-RIVS-free probability over time and the number at risk in the palliative care patients treated with cetuximab. CR-RIVS, chemoport-related right innominate vein stenosis; Tx., treatment.

The CR-RIVS-free probability was significantly lower in the patients who received palliative chemotherapy than in those who received adjuvant chemotherapy (*p* < 0.001) (Fig 5). In the subgroup analysis of patients receiving palliative therapy, the log-rank tests revealed that those treated with bevacizumab exhibited a lower CR-RIVS-free probability compared to that in those who did not receive bevacizumab during palliative treatment (*p* < 0.001) (Fig 6a). In contrast, the log-rank tests revealed no significant difference in the risk of CR-RIVS between patients treated with and without cetuximab (*p* = 0.91) (Fig 6b).

## Discussion

This study found a CR-RIVS prevalence of 6.6% (95% CI: 5.4%–8.0%) in patients with CRC. This finding aligns with previous studies that reported venous stenosis rates of approximately 7% in patients with peripherally inserted central catheters or chemoports [5,9].

Innominate vein stenosis can lead to several potentially severe complications that may affect patient comfort and diagnostic accuracy. While innominate vein stenosis is often asymptomatic owing to the compensatory function of collateral channels, some patients may experience ipsilateral swelling of the arms, venous distension, and, in severe cases, upper extremity edema accompanied by pain and discomfort, skin ulceration, and recurrent infection. These complications may increase the likelihood of requiring additional invasive procedures, further exacerbating patient discomfort and the risk of experiencing additional adverse effects [19]. Furthermore, vascular access failure and chemoport malfunction may occur, which can complicate the timely administration of necessary treatments [6,20].

From a radiological perspective, the use of contrast media plays an important role in enhancing image quality and facilitating lesion detection in chest CT scans [21,22]; however, innominate vein stenosis can impede the proper administration of contrast media, thereby negatively affecting the quality of imaging. Stenosis can also lead to various imaging artifacts and abnormalities that compromise the diagnostic accuracy and complicate interpretation; for example, one of the primary effects of innominate vein stenosis on image quality is the occurrence of perivenous artifacts, which manifest as streak-like distortions around the affected vessels that obscure adjacent anatomical structures and potentially mask the presence of pathological indicators [7]. This limitation is particularly crucial in patients with cancer for whom the precise evaluation of potential metastatic progression or treatment responses is essential. Artifacts and abnormal contrast distribution can obscure small lesions or lead to false-positive findings, potentially affecting clinical decision-making. In addition, the venous reflux induced by stenosis can result in suboptimal contrast enhancement and vascular opacification [7], and the effects may depend on laterality. For example, Hingwala et al. demonstrated that left-sided contrast injections were more likely to be affected by venous reflux owing to anatomical factors, such as compression of the left innominate vein between the sternum and aortic arch, leading to suboptimal opacification of target vessels in magnetic resonance angiography [23]. A particularly concerning consequence of venous reflux is the vanishing bone metastasis phenomenon in which contrast reflux into the vertebral body can create focal areas of high attenuation that mimic sclerotic bone metastases. Shin et al. reported that these high-density lesions had a mean attenuation of 784 Hounsfield units, which overlaps with the range observed for sclerotic bone metastases [9,24,25]. This similarity in appearance can lead to misinterpretation by radiologists, potentially resulting in unnecessary additional diagnostic work-ups, increased patient anxiety, and inflated healthcare costs. Awareness of the potential for innominate vein stenosis and recognition of its imaging manifestations can help radiologists take preventive measures, such as injecting contrast media on the side contralateral to that of the chemoport.

To effectively address the complications and degradation of image quality caused by CR-RIVS, understanding its underlying mechanisms is essential. Endothelial wall damage and inflammation caused during catheter placement as well as chemotherapeutic agent infusion can lead to thrombus formation around the chemoport, which undergoes partial lysis and recanalization over time and can result in thickening of the venous wall and subsequent venous stenosis [10]. Furthermore, the organization of the residual thrombus can lead to stenosis or chronic venous occlusion [5]. This pathophysiological mechanism suggests a potential association may exist between the risk factors for chemoport-induced thrombosis and the occurrence of innominate vein stenosis. Patients with cancer who are undergoing chemotherapy have a heightened susceptibility to developing a venous thromboembolism owing to various factors, including the hypercoagulable state induced by both malignancy and chemotherapeutic agent administration [26]. For instance, certain chemotherapeutic regimens, particularly those involving fluorouracil, have been identified as independent risk factors for peripherally inserted central catheter-related thrombosis [27]. Bevacizumab, a monoclonal antibody that targets vascular endothelial growth factor, has been implicated in the increased risk of thrombosis, and animal studies have demonstrated that vascular endothelial growth factor inhibition can lead to endothelial cell apoptosis and the exposure of procoagulant phospholipids on the luminal surface of blood vessels, thereby resulting in an enhanced prothrombotic environment [28]. Although the relationship between bevacizumab treatment and systemic venous thrombosis in clinical settings is still debated [26,29–31], Kim et al. reported an association between chemoport-related venous thrombosis and bevacizumab use [14].

In the present study, the CART decision tree analysis identified treatment in the palliative setting as the key partitioning factor for CR-RIVS. However, since bevacizumab is exclusively administered in the palliative setting, these two variables are highly collinear, and the palliative setting variable likely serves as a surrogate for bevacizumab exposure. In the multivariable Cox proportional hazards analysis, age exhibited a modest but significant association with increased risk (HR = 1.008, *p* = 0.002), and the time×palliative treatment interaction (HR = 2.820 × 10^−3^, *p* < 0.001) and palliative treatment setting (HR = 2.760 × 10^10^, *p* < 0.001) emerged as highly significant predictors. The significant negative correlation coefficient of the time×palliative treatment interaction aligned with the patterns of the density plots, with the highest event density occurring approximately 12 months post-chemoport insertion, with a subsequent gradual decline over time and across bevacizumab treatment cycles. This temporal pattern, which was supported by both the negative time×palliative treatment interaction and density plots, suggests the CR-RIVS risk is not cumulative over time and is concentrated in the early interventional period. The CR-RIVS-free probability was significantly lower in patients receiving palliative chemotherapy than in those receiving adjuvant chemotherapy (*p* < 0.001), which was consistent with the results of the CART and multivariable Cox proportional hazards analyses. In the subgroup analysis of patients receiving palliative chemotherapy, those treated with bevacizumab exhibited a lower CR-RIVS-free probability (*p* < 0.001), whereas no significant difference was observed in those treated with cetuximab (*p* = 0.91). The Kaplan-Meier curves clearly illustrated this distinction, with bevacizumab-treated patients experiencing earlier and more frequent CR-RIVS events, which is consistent with previous studies that identified bevacizumab as a risk factor for both chemoport-related thrombosis [14] and vanishing bone metastasis [9]. This observation is particularly noteworthy given that both agents are commonly used in palliative treatment regimens for CRC; however, only bevacizumab appeared to significantly impact CR-RIVS-free probability. Considering the endothelial damage that may have occurred during chemoport insertion and the initial phase of chemotherapeutic agent infusion, the difference between the two antibodies may be indicative of an association between bevacizumab and CR-RIVS occurrence, through its anti-VEGF mechanism.

This study had some limitations. First, this was a single-center retrospective study. Second, this study only evaluated CR-RIVS, as it focused on the most common side for chemoport catheter placement, which may limit the applicability of the findings to other venous structures that may have resulted in systemic thromboembolic events. Third, the definition of CR-RIVS may be somewhat subjective, potentially leading to interobserver variability. Fourth, as the interval between chest CT scans varied by patient, venous stenosis could have developed before detection in chest CT images, which could have affected the accuracy of the time-based analyses. This difference in CT follow-up frequency between palliative and adjuvant settings may introduce surveillance bias. However, the median observation period was shorter in the palliative group (978 days) than in the adjuvant group (1,676 days), yet CR-RIVS events were more frequent in the palliative group, suggesting that the effect of surveillance bias on the overall direction of the findings is likely limited. Fifth, this study did not focus on the biological profile of the tumors, such as the presence of Kirsten rat sarcoma virus or epidermal growth factor receptor mutations, which may act as confounders. Sixth, although chemoport removal or exchange was observed in 10.5% of CR-RIVS patients, this study did not comprehensively assess all clinical consequences of CR-RIVS, such as rates of symptomatic stenosis or treatment delays. Future prospective studies should evaluate the full clinical impact of CR-RIVS on patient outcomes and treatment continuity.

In summary, the prevalence of CR-RIVS in patients with CRC was 6.6%, and bevacizumab use was identified as the factor most strongly associated with CR-RIVS occurrence. This study underscores the importance of heightened awareness for CR-RIVS in patients with CRC who receive bevacizumab-containing regimens through chemoports and highlights the need for appropriate monitoring in this population.
